# Real-time surgical tool tracking and pose estimation using a hybrid cylindrical marker

**DOI:** 10.1007/s11548-017-1558-9

**Published:** 2017-03-24

**Authors:** Lin Zhang, Menglong Ye, Po-Ling Chan, Guang-Zhong Yang

**Affiliations:** Hamlyn Centre for Robotic Surgery, Imperial College London, London, UK

**Keywords:** Surgical tool, Tracking, Pose estimation, Cylindrical marker, Image guidance

## Abstract

***Purpose*:**

To provide an integrated visualisation of intraoperative ultrasound and endoscopic images to facilitate intraoperative guidance, real-time tracking of the ultrasound probe is required. State-of-the-art methods are suitable for planar targets while most of the laparoscopic ultrasound probes are cylindrical objects. A tracking framework for cylindrical objects with a large work space will improve the usability of the intraoperative ultrasound guidance.

***Methods*:**

A hybrid marker design that combines circular dots and chessboard vertices is proposed for facilitating tracking cylindrical tools. The circular dots placed over the curved surface are used for pose estimation. The chessboard vertices are employed to provide additional information for resolving the ambiguous pose problem due to the use of planar model points under a monocular camera. Furthermore, temporal information between consecutive images is considered to minimise tracking failures with real-time computational performance.

***Results*:**

Detailed validation confirms that our hybrid marker provides a large working space for different tool sizes (6–14 mm in diameter). The tracking framework allows translational movements between 40 and 185 mm along the depth direction and rotational motion around three local orthogonal axes up to $\pm 80^\circ$. Comparative studies with the current state of the art confirm that our approach outperforms existing methods by providing nearly 100% detection rates and accurate pose estimation with mean errors of 2.8 mm and 0.72$^\circ$. The tracking algorithm runs at 20 frames per second for $960\times 540$ image resolution videos.

***Conclusion*:**

Experiments show that the proposed hybrid marker can be applied to a wide range of surgical tools with superior detection rates and pose estimation accuracies. Both the qualitative and quantitative results demonstrate that our framework can be used not only for assisting intraoperative ultrasound guidance but also for tracking general surgical tools in MIS.

## Introduction

Minimally invasive surgery (MIS) is becoming a standard procedure for a range of surgical disciplines. Advantages of MIS include less blood loss, less post-operative pain, lower infection rates and shorter hospitalisation [1]. However, there are intrinsic limitations to existing MIS approaches, including a loss of direct organ manipulation, lack of dexterity via ‘keyhole’ access and poor depth perception using a monocular laparoscope. To identify the anatomical structures such as vessels and tumours, intraoperative ultrasound has been used [2]. However, a well-known drawback of using intraoperative ultrasound is that surgeons need to interpret the ultrasound images and perceptually relate these images to the operative scene captured by a laparoscope [3]. This is particularly challenging, as the ultrasound images and laparoscopic videos are displayed separately on different screens. To reduce the mental workload of a surgeon, a previous study presented in [2] has introduced an intuitive visualisation method, which is achieved by registering the 2D ultrasound images into the surgical scene. This approach enables surgeons to observe details from both ultrasound and laparoscope in a single display. To provide consistent image overlays, the ultrasound probe needs to be tracked during operation.

A direct use of laparoscopic images for tracking instrument in MIS is more practical than of other methods where extra tracking devices [4] are used. The introduction of additional tracking devices into the theatre would not only occupy the valuable space in the operating field, but also suffer from intrinsic limitations of these tracking devices such as ferromagnetic interference and line-of-sight issues. Previous methods have extracted 2D locations of a planar [5] or cylindrical [6] marker that is rigidly attached to the probe. In Table 1, a list of marker-based tracking methods for intraoperative ultrasound probes is presented. Among these methods, pose estimation is treated as a Perspective-n-Point (PnP) problem [7]. However, pose estimation of a planar marker may provide two ambiguous solutions where the incorrect one should be eliminated [8].

**Fig. 1:**
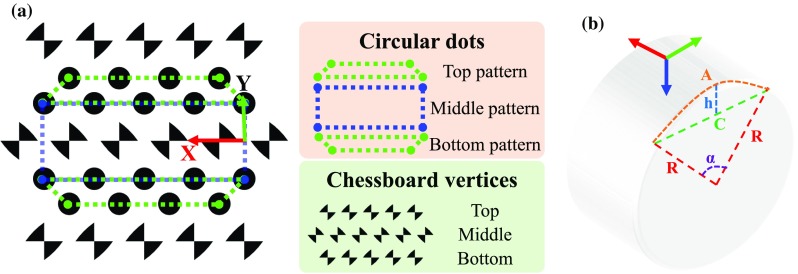
**a** A *top view* of the proposed marker for cylindrical object tracking. The hybrid marker consists of *circular-dot* patterns and chessboard vertices. **b** A *side view* of a cylinder showing related parameters to define a marker’s local coordinate frame

In this paper, we propose a new marker design for cylindrical surgical tools. The marker consists of three patterns of circular dots and lines of chessboard vertices, as shown in Fig. 1. The circular-dot pattern is used to estimate the pose of the tool while the chessboard vertices are used to eliminate the ambiguity in pose estimation. To further improve detection rates, a tracking component considering temporal information is employed to cater for failures in marker detection. The proposed tracking framework is evaluated with various surgical tools including ultrasound probes and robotic instruments. Detailed validation is provided, and comparison results demonstrate our framework outperforms other alternative approaches, both in detection rates, work space coverage and pose estimation accuracies.

**Table 1:** Comparison of visual marker-based tracking methods for intraoperative ultrasound probe

Method	Probe shape	Pattern type	Robotic/freehand	Real time
[9]	Planar	Chessboard	Freehand	Yes
[10]	Planar	Chessboard	Robotic	No
[5]	Planar	Circular dot	Robotic	Yes
[6]	Cylindrical	Chessboard	Freehand	No
Ours	Cylindrical	Hybrid	Both	Yes

## Methods and materials

### Design of a hybrid marker

We propose a novel hybrid marker including both circular dots and chessboard vertices for a cylindrical object as shown in Fig. 1a. The circular dots can be labelled into three groups denoted as top, middle and bottom patterns according to their relative location. Each pattern has two lines of circular dots which are used for pose estimation. Top and bottom patterns are vertically asymmetric, and the middle pattern is symmetric such that adjacent patterns can be easily differentiated. In addition, we placed three lines of chessboard vertices between the circular-dot patterns. The middle vertices have a 90$^\circ$ orientation shift relative to the top/bottom vertices. These vertices are used to remove ambiguous “Pose estimation” section. The marker can be horizontally placed on a cylindrical object such that lines on the pattern are parallel to the axial axis of a cylindrical tool. We define a local coordinate frame of a cylinder, as shown in Fig. 1b, where several parameters can be defined:

1 $$\begin{aligned} A&= \alpha R \nonumber \\ C&= 2R\sin \frac{\alpha }{2}\nonumber \\ h&= R\left( 1-\cos \frac{\alpha }{2}\right) . \end{aligned}$$

Here *A* is the arc length when the angle and radius are $\alpha$ and *R*, respectively. *C* is the chord length and *h* is the corresponding segment height.

Given a marker position $p=[x,y]^T$ located in a 2D coordinate frame as indicated in Fig. 1a, its corresponding 3D position $P=[X, Y, Z]^T$ in the local coordinate frame of the cylinder can be defined by using Eq. 1:

2 $$\begin{aligned} X&= x \nonumber \\ Y&= R \sin \frac{y}{R} \nonumber \\ Z&={\left\{ \begin{array}{ll} R - \sqrt{R^2-Y^2} &{} \text {if } y \le \frac{\pi R}{2} \\ R + \sqrt{R^2-Y^2} &{} \text {if } y > \frac{\pi R}{2} \end{array}\right. }. \end{aligned}$$

These points will be used as model points for the marker “Pose estimation” section.

### Feature detection

**Fig. 2:**
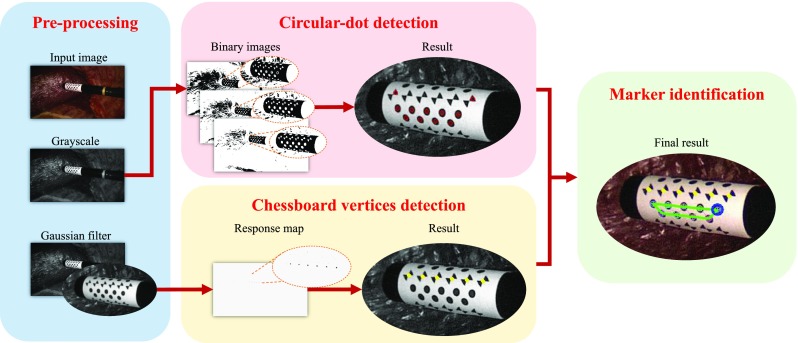
Algorithm workflow of the marker detection algorithm. The binary images are generated using threshold values between 70 and 100 with an interval of 10. The detected *dots* are *circled* in *red*, and the chessboard vertices are labelled in *yellow*. The *first dot* in the pattern is *circled* in *blue*

A workflow of the detection algorithm for the proposed marker is shown in Fig. 2. At each iteration, a greyscale image is obtained from a colour image and used for circular-dot detection. The greyscale image is then used to generate multiple binary images, as shown in Fig. 2, based on thresholding. The use of multiple binary images is effective for discarding blobs that are false positives, as they would not consistently appear when varying the threshold values. The threshold values should be determined according to the lighting condition of the scene. In our experiments, we used a set of thresholds (70–100) with an interval of 10, which is a good trade-off between speed and accuracies. To locate circular dots, we apply contour tracing on the binary images to extract circular blobs. In this paper, we exploit [11] in our implementation^1^ to extract the circular blobs.

Several criteria were used to filter spurious blobs which do not belong to the marker. A blob is removed if its area is not in line with a desired value which can be defined based on the possible minimal and maximal size of a blob in the image. In addition, the convexity of a blob is also used because the perspective projection of a circular dot on an image results in a convex shape, whereby non-convex blobs can be removed. We define the convexity as the ratio of the blob’s area to the area of the blob’s convex hull. Another adopted criterion for removing polygonal blobs is the circularity which is defined as:

3 $$\begin{aligned} O = \frac{4 \cdot \pi \cdot E}{P^{2}}, \end{aligned}$$

where *E* and *P* are the area and perimeter of the blob, respectively. Lastly, a blob which belongs to the marker should appear at least *N* times in the binary images, where *N* is called repeatability. The best numbers for area, convexity, circularity and repeatability are 50–2000, 0.85, 0.75 and 2.

The chessboard vertices are detected using an efficient detector [13] designed for chessboard corner feature. As shown in Fig. 2, a Gaussian filter is applied to the greyscale image to remove speckles and noises that might degrade the performance of the chessboard detector. For each pixel in the filtered image, a ring of 16 pixels around the pixel are sampled at a constant radius with equal angular spacing. A response map is calculated using the sampled pixels, which is shown in Fig. 2, and a chessboard feature belongs to the marker if its value in the response map is greater than a threshold value. We follow the method in [13] to calculate the response map. Specifically, an overall response for each pixel equals to a sum response subtracting a difference response and mean response. The sum response calculates the summation of opposite samples. The difference response is defined as the difference of opposite samples. The mean difference is an absolute difference of a local intensity mean and a larger spatial mean. To further remove spurious features, which tend to have low responses, non-maximum suppression [14] is applied to retrieve the location of peak responses. In addition to offering the position information, the chessboard detector also provides the orientation of a vertex in the image coordinate using 1 of 8 labels. Given the orientation information, a spurious feature can be removed as its orientation label will not in accord with the desired one which will be described in “Marker identification” section.

**Fig. 3:**
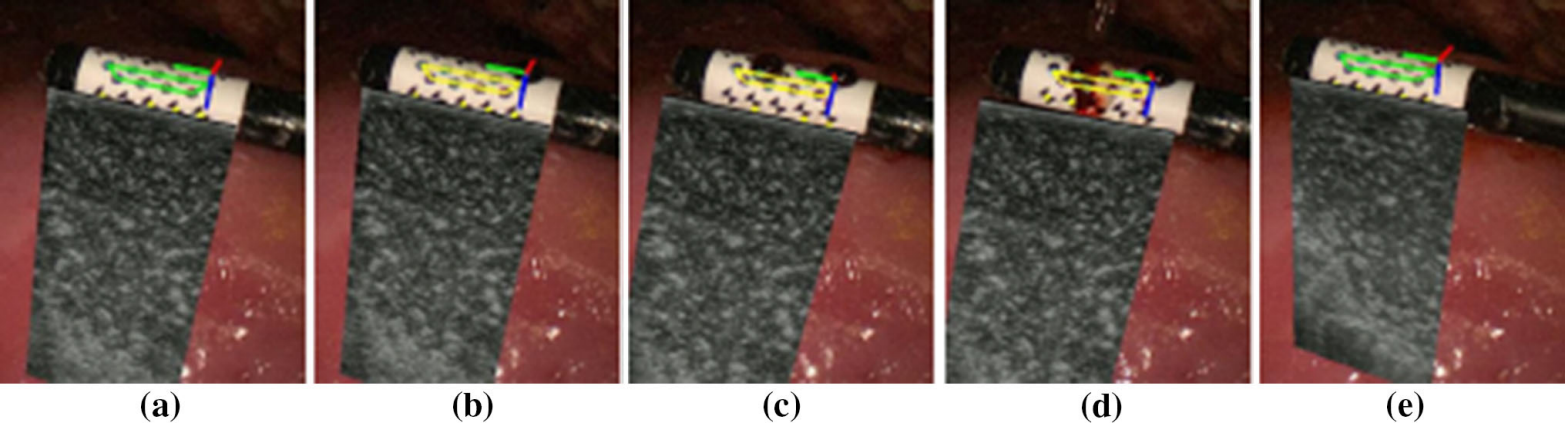
A sequence of images showing how the tracking algorithm reacts when the marker is partially occluded by fake blood. The *green outline* of the marker indicates successful detection while *yellow outline* means detection failure and tracking component is used

Furthermore, triangle forming the chessboard vertices may be detected as a dot. Therefore, the detected chessboard features are also used to reject the false detection of a circular dot.

### Marker identification

To estimate the marker pose, the correspondence information of the detected dots and the model points is required. We have used the following cues. Firstly, dots that are close to each other are assigned to the same cluster. The cluster that has the largest number of dots is identified as the marker. Secondly, four endpoints locating at corners are found by using the convex hull of the cluster and the corners should have sharp angles. The endpoints are sorted based on the fact that two long edges are parallel, and then, the model circular-dot pattern can be transformed to the image using correspondences of the sorted endpoints. Thirdly, the identity of each detected dot is found by using the closest point in the transformed model pattern. Since the top and bottom patterns are the same, but vertically flipped, if an asymmetric pattern is detected, we need to decide which pattern it belongs to. Typically, surgical instrument movements are constrained by the incision point, so it is assumed that the pattern cannot be flipped horizontally. Since the circular dots in the detected pattern are identified, if the first dot of the pattern (as depicted in Fig. 2) is on the left side of the last dot, the pattern is a top pattern; otherwise, it is a bottom pattern. Finally, the orientation of the symmetric (middle) pattern can be determined similarly by looking at the relative position of the dots. By having the labelled circular-dot pattern, the chessboard vertices can be identified as well. The relative orientation between the chessboard vertices and the circular-dot pattern is fixed; therefore, the desired orientation of the vertices can be deduced from the orientation of the circular-dot pattern.

### Tracking

In practice, occlusion can occur due to rotational motion of the probe as well as blood stains on the marker, all of which would result in missing dots during marker detection. To address this problem, we compute a homography $\mathtt {H}$, which is a $3 \times 3$ matrix with 8 DoF, representing perspective transformation between two planes. The circular-dot pattern is defined in a reference coordinate frame where position of the dots is denoted as $p_{r}$. During tracking, the projection of the circular dots on the camera image plane is denoted $p_{m}$. The homography links them via: $p_{m} = \mathtt {H} p_{r}$.

If the detection component of the framework is not able to detect the marker, a sparse optical flow method [15] is applied, considering the temporal information, to find the current position of visible dots. The dots are used to estimate the homography $\mathtt {H}$. This homography can be estimated using only four pairs of non-collinear points, thus robust to occlusion. The position of a missing dot can be calculated from its correspondence in the reference coordinate frame by using the homography.

**Fig. 4:**
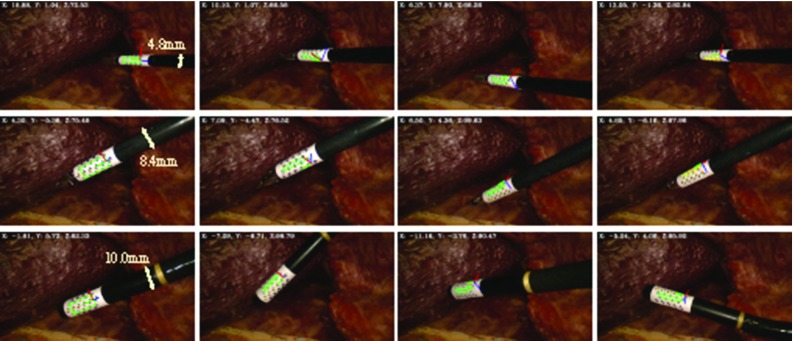
Qualitative results of the tracking framework for the proposed marker on various types of surgical tool. A 5-mm monopolar cautery (*top row*), a da Vinci large needle driver (*middle row*) and a laparoscopic ultrasound probe (*bottom row*) are tracked, respectively. The coordinate axis in *red*, *green* and *blue* shows that the pose has been correctly estimated

An illustration of how the tracking component deals with the occlusion is shown in Fig. 3. The marker is detected correctly at the beginning. As blood is stained onto the probe, which is shown in Fig. 3b–d, the detection method cannot extract the marker’s location and the tracking method is used. The tracking method, on the other hand, is able to locate the marker correctly until a major area of the pattern has been occluded, as shown in Fig. 3d. In this case, the tracking results start drifting which leads to wrong pose estimation. In Fig. 3e, detection of the marker is recovered when the blood stain is cleaned. For self-occlusion, as shown in Fig. 4, the algorithm can deal with large rotational motion where the marker is partially visible.

### Pose estimation

**Fig. 5:**
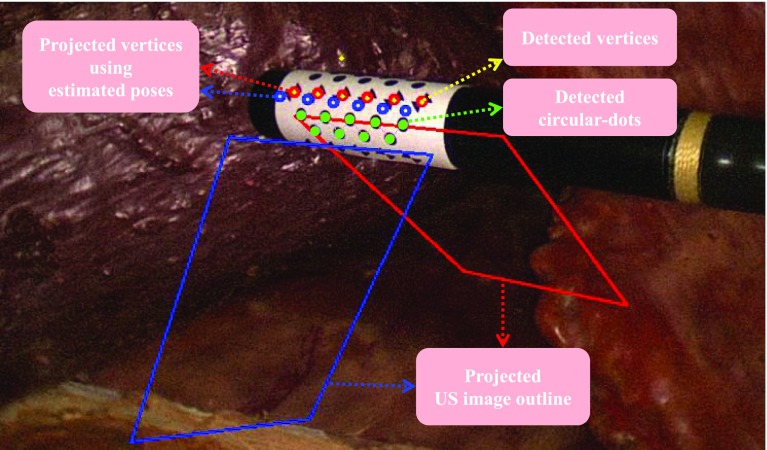
An illustration of ambiguous pose removal using chessboard vertices. The *red* and *blue circles* indicate projections of the model chessboard vertices using two candidate poses. The two quadrangles represent the projected outline of an ultrasound image on the image. In this example, we can easily see that the pose corresponds to the *red* is correct

Given the model points defined in the marker’s local coordinate frame and their corresponding tracked projections on an image of a calibrated camera, the marker pose can be estimated via PnP methods. However, a planar model has the rotation ambiguity which corresponds to a reflection of the points about the plane whose normal points along a line of sight pass through the points [8]. The pose estimation problem becomes ambiguous when the projection of the model is close to affine, which typically occurs if the pattern is either small or far from the camera. We employed [8], which is particularly useful as it provides two solutions that can correctly align the projections using the estimated pose with the detected correspondences. One of the solutions should be eliminated when ambiguity occurs. Normally, the correct solution will result in a smaller re-projection error, which represents the difference between the projections and tracked dots. In each image, the re-projection errors from both solutions are compared, and if both give small errors close to the zero, the ambiguity occurs. To resolve this issue, we use points from a different plane where the wrong solution gives a large re-projection error. An ambiguous case is shown in Fig. 5 in which two marker poses are estimated using the detected circular dots indicated in green. If we project the circular-dot pattern using the two estimated poses, then both projections will be aligned with the tracked pattern. Therefore, we take advantage of the chessboard vertices on the hybrid marker which are not located in the same plane as the circular-dot pattern. In Fig. 5, projected vertices using the two poses are shown in red and blue, respectively. They are compared to the current vertices detection, which is drawn as yellow dots, and the closer one (smaller re-projection error) is chosen as the correct pose. In this example, the pose corresponds to the red projections which is correct. To verify this, we place an ultrasound image locating at the transducer of the probe and then project the outline of the ultrasound image on the camera image using the estimated poses. As shown in Fig. 5, it is clear that the red projected outline is the correct one.

**Fig. 6:**
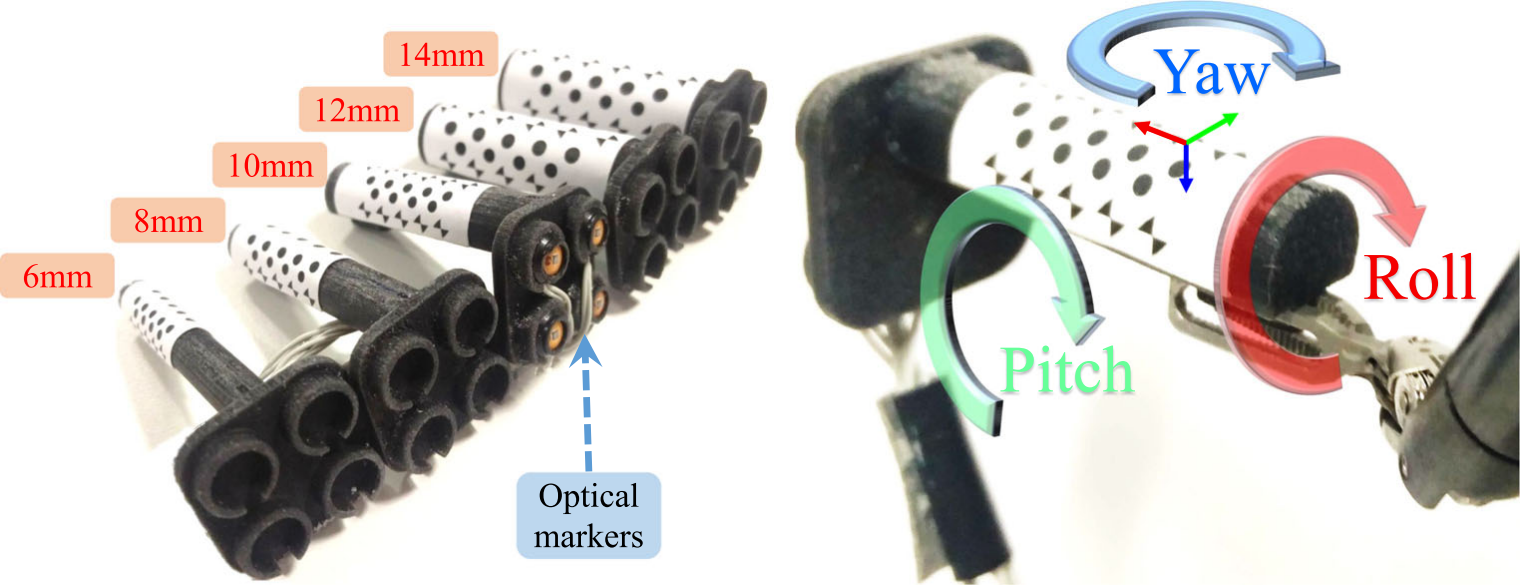
3D printed rigid body with various sizes for validation (*left*) and definition of the marker’s local coordinate system (*right*)

## Experimental results

### Hardware set-up

**Fig. 7:**
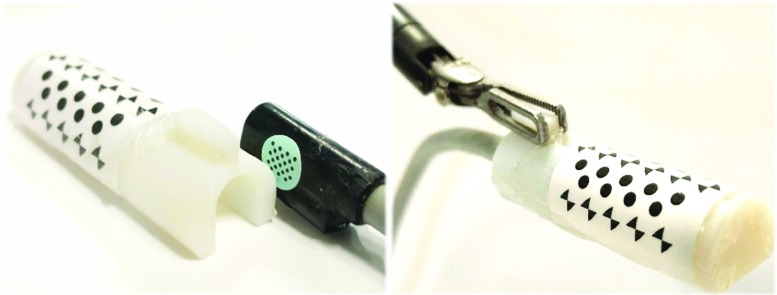
An housing adaptor that can hold a planar ultrasound probe. With the housing, the planar probe can be tracked by using the proposed marker

Images of surgical view are captured using a 10-mm-diameter monocular laparoscope (Karl Storz GmbH, Germany) whose intrinsic parameters are calibrated using [16]. We tested the proposed framework on two ultrasound transducers: (a) UST-533 linear array microsurgery probe and (b) UST-5550 linear array two-way laparoscopic probe. Both transducers are driven by a Prosound $\alpha$-10 machine (Hitachi Aloka Medical Ltd, Japan). Laparoscopic and ultrasound images are streamed to a computer (3.4 GHz CPU, 16G RAM) using DVI2USB converters (Epiphan System Inc, Canada). To validate the robustness of the tracking framework on the proposed cylindrical marker, five cylindrical rigid bodies with various diameters from 6 to 14 mm are designed and printed, as shown in Fig. 6. Four active optical sensors are placed on the body which is tracked by an Optotrak Certus system (Northern Digital Inc, Canada) that achieves 0.1 mm accuracy. The rigid body can be grasped and moved by a da Vinci Cadiere forceps instrument (Intuitive Surgical, USA). As the UST-533 probe does not have a cylindrical shape, a housing adaptor is printed, as shown in Fig. 7, so that it can be tracked by using the proposed marker.

### Work space analysis

To identify the work space of the proposed marker and associated detection algorithm, we recorded the maximal distance and rotation of the tool, above which the marker cannot be detected. We investigated the translational motion along camera’s optical axis and the rotational motion around the local axis (roll, pitch and yaw) of the validation rigid body as depicted in Fig. 6. The dimension of the marker has been modified based on the size configuration in order to maximise the marker’s coverage on the curved surface. For testing the rotational motion, the marker was placed about half of its maximal work distance to obtain better visibility. We repeated the experiment on all five markers with different sizes whose results are shown in Table 2. A positive correlation can be found between the marker size and distance to the camera. As the marker becomes larger, the maximal work distance is accordingly extended due to better visibility. We observed that a reasonable work space for most minimally invasive procedures ranges from 50 to 200 mm. The rotation around the roll axis has also been extended up to $\pm$89$^\circ$ when the tool diameter increases to 14 mm. Regarding rotation around the pitch and yaw axis, the marker is capable of dealing with rotation more than $\pm$70$^\circ$ and $\pm$80$^\circ$, respectively. In summary, these results show that the proposed marker can be applied to instruments with different size while providing a large work space.

**Table 2:** Work space for the proposed marker on various tool sizes

Diameter (mm)	Distance to camera (mm)	Roll ($^\circ$)	Pitch ($^\circ$)	Yaw ($^\circ$)
6	30–125	±68	±56	±81
8	35–160	±76	±71	±83
10	40–185	±82	±76	±85
12	60–200	±85	±78	±83
14	60–210	±89	±78	±80

**Fig. 8:**
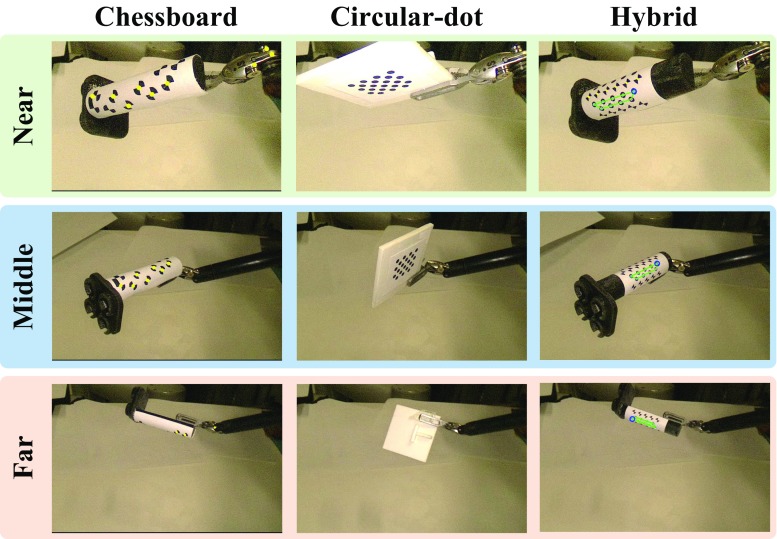
A snapshot of the experiment for detection rate comparison. For each distance group, the same movement has been reproduced to test the detection performance of different markers. The *circular-dot* marker cannot be detected while the maker is in a very *slant view*. In the far distance group, we show a scenario where the marker is rotated around its axial axis, so only the other side is visible

### Detection rates

As reported in [5], the detection rate of a circular-dot feature is higher than of a chessboard feature. In order to evaluate the detection rate of the proposed marker, which consists of both circular dot and chessboard vertices, we compared with two previous marker designs with one containing only chessboard vertices [6] and the other one containing only circular-dot [5]. The proposed hybrid and the chessboard only markers were placed on the 10-mm-diameter cylindrical rigid body. The circular-dot marker can only be placed on a planar surface due to the limitation of its detection algorithm. The size of the circular-dot marker was set to be similar to our hybrid marker for a fair comparison. The experiment was divided into three groups based on different distances to the camera: near (50–100 mm), middle (100–150 mm) and far (150–200 mm), as shown in Fig. 8. For each group, we run three trials for the three types of markers using the same trajectory which mainly consists of rotational motion around the roll, pitch and yaw axis. The illumination level was fixed in the same distance group. The trajectory is firstly recorded from a manual demonstration using the da Vinci robot with dVRK controllers [17]. During the trial, the recorded trajectory is played back and the detection results are saved. For the chessboard vertices marker, a detection result is considered as a success if at least four features are found as required by [6]. For the circular-dot marker, we only apply the detection part without tracking so that the performance is similar to [5]. In this case, a frame is accepted if the number of detected blobs equals to the number of dots in the marker. For circular dot in the hybrid marker, one is considered to be a success if either of the top, middle or bottom pattern is detected.

The quantitative results, as shown in Table 3, confirm that the hybrid marker with proposed tracking framework is superior to previous methods in terms of marker detection rate. In particular, the circular-dot pattern(s) in the hybrid marker were nearly 100% detected in different distances. The planar circular-dot marker has much less detection rate due to self-occlusion caused by the rotational motion. Regarding the chessboard feature, the results based on the hybrid marker are comparable to the method introduced in [6] for near and middle distance. However, our chessboard feature detection method can be deteriorated when the marker is far away. This is because more rigorous criteria were used to ensure the correspondence between the detected chessboard features and model points for accurate pose estimation. For example, if less than four chessboard features with the same orientation are detected, the result is regarded as a failure.

**Table 3:** Detection rates of different markers in different work distances

Distance	Chessboard vertices [6] (%)	Circular dot [5] (%)	Hybrid chessboard (ours) (%)	Hybrid dots (ours) (%)
Near (50–100 mm)	87.2	53.2	98.3	99.7
Middle (100–150 mm)	86.8	61.5	84.7	100
Far (150–200 mm)	79.5	75.7	11.0	99.8

### Pose estimation error

In order to quantitatively validate the pose estimation error, we used the Optotrak Certus system to obtain the ground truth. To this end, two registrations are required: optical sensors to the hybrid marker $\mathbf {T}_{S}^{M}$ and optical system to laparoscope $\mathbf {T}_{O}^{L}$. The problem can be treated as a $AX=YB$ problem which is defined as:

4 $$\begin{aligned} \mathbf {T}_{O}^{S} \cdot \mathbf {T}_{S}^{M} = \mathbf {T}_{O}^{L} \cdot \mathbf {T}_{L}^{M}, \end{aligned}$$

where $\mathbf {T}_{O}^{S}$ and $\mathbf {T}_{L}^{M}$ are optical sensors pose in the optical system coordinate frame and marker pose in laparoscope coordinate frame, respectively. A total of 10 $\mathbf {T}_{O}^{S}$ and $\mathbf {T}_{L}^{M}$ were acquired, so that $\mathbf {T}_{S}^{M}$ and $\mathbf {T}_{O}^{L}$ can be calculated using [18].

**Table 4:** Pose estimation error for the proposed marker on different tool size

Diameter (mm)	Translation mean error $\pm$ STD (mm)	Rotation mean error $\pm$ STD ($^\circ$)
6	1.43 $\pm$ 1.09	0.55 $\pm$ 0.38
8	4.15 $\pm$ 1.53	1.70 $\pm$ 0.47
10	2.80 $\pm$ 2.02	0.72 $\pm$ 0.52
12	2.53 $\pm$ 1.40	0.69 $\pm$ 0.33
14	3.79 $\pm$ 1.70	0.90 $\pm$ 0.45

**Fig. 9:**
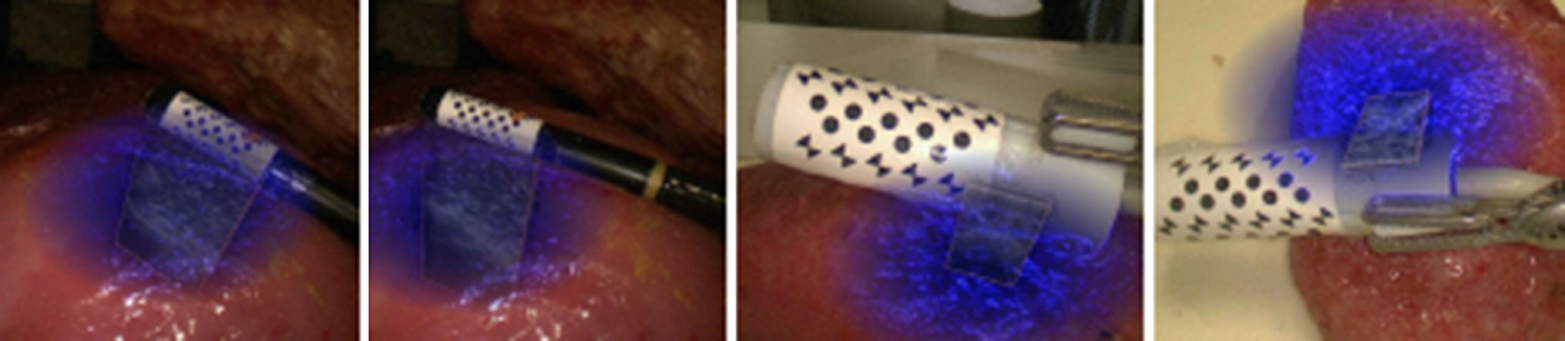
Ultrasound image is fused with laparoscopic image using an inverse realism technique [19]

For experimental validation, we ran five trials for the markers with different diameters. For each trial, a total of 50 measurements were made near half of the maximum work distance (as defined in Table 2). For each measurement, a relative pose between the estimated and ground truth pose is calculated as:

5 $$\begin{aligned} \mathbf {T}_{M}^{M^{\star }} = {\mathbf {T}_{L}^{M}}^{-1} \cdot {\mathbf {T}_{O}^{L}}^{-1} \cdot \mathbf {T}_{O}^{S} \cdot \mathbf {T}_{S}^{M^{\star }}, \end{aligned}$$

where $\mathbf {T}_{S}^{M^{\star }}\,=\,\mathbf {T}_{S}^{M}$ is the calibrated transformation between the optical sensors and hybrid marker. The pose estimation error, as shown in Table 4, is the translation and rotation component of $\mathbf {T}_{M}^{M^{\star }}$. It is worth noting that this error combines the registration error of $\mathbf {T}_{S}^{M}$ and $\mathbf {T}_{O}^{L}$ in Eq. 4. The results in Table 4 show that the pose estimation error is slightly smaller than results presented in [6] where an error of $4.4 \pm 3.3$ mm was reported. It is worth noting that the 8-mm tool performed worse than the 6 mm one due to the marker design. For the 6-mm marker, the top and bottom chessboard vertices are closer than the 8-mm marker, so they can be detected more frequently, as shown in Fig. 6. To improve detectability of the 8-mm marker, the pattern design can be adjusted so that at least one line of chessboard vertices is visible.

### Example application to ultrasound overlay

Here, an example application of the proposed tracking approach for surgical AR is demonstrated. To this end, we combine our tracking framework with an AR approach introduced in [19]. As shown in Fig. 9, the proposed framework provides accurate tracking, which enables seamless integration intraoperatively with see-through vision. The speed including detection and tracking is 20 frames per second on $960\times 540$ images. The visualisation with inverse realism can be processed in real time [20].

### Discussion

The proposed tracking framework allows for large rotational and translational motion while providing superior detection rate and pose estimation accuracy. These are shown in Tables 2, 3 and 4. In comparison with the previous methods [5, 6], the proposed framework improves the detection rate by considering the temporal information between consecutive images. Further, the proposed method achieves competitive pose estimation accuracy compared to [6] while allowing real-time processing which enhances its usability for clinical applications. The current framework can cope with self- and partial occlusion as demonstrated in Fig. 3. It is worth noting that the effect of smoke is not yet considered. This will be included in our future work. We have also shown that the proposed marker can be applied to different types of surgical instruments as shown in Fig. 4.

Currently, the camera calibration is carried out offline and the parameters are assumed to be fixed during tracking. A useful improvement in the framework is to include simultaneous camera calibration as the marker is tracked. This will enhance the applicability of the framework in clinical use where camera calibration may be troublesome when camera focus has to be changed.

## Conclusion

In conclusion, a novel hybrid marker made of circular dots and chessboard vertices has been proposed, along with an algorithm for tracking surgical tools. The circular-dot pattern is used for pose estimation while chessboard vertices are used to address the issue of ambiguous pose estimation. In addition, temporal information is considered to deal with partial occlusion. Extensive experiments have been conducted on tracking a cylindrical ultrasound probe with different scenarios. Both qualitative and quantitative results confirm that our framework outperforms the current state-of-the-art approaches in terms of detection rates and pose accuracies, while also provides a large work space. Furthermore, we also show the results of our framework on different surgical tools with varying sizes, and the results verify the practical value of our framework in minimally invasive procedures.

## Footnotes

1 An implementation of OpenCV is adopted [12].
